# A Human Gonadal Cell Model From Induced Pluripotent Stem Cells

**DOI:** 10.3389/fgene.2018.00498

**Published:** 2018-10-24

**Authors:** Daniel Rodríguez Gutiérrez, Wassim Eid, Anna Biason-Lauber

**Affiliations:** ^1^Section of Medicine, Endocrinology Division, University of Fribourg, Fribourg, Switzerland; ^2^Department of Biochemistry, Medical Research Institute, University of Alexandria, Alexandria, Egypt

**Keywords:** Sertoli cells, iPSC, NGS, reprograming, cell model, disorders/differences of sex development, DSD

## Abstract

Sertoli cells are main players in the male gonads development and their study may shed light on 46,XY disorders of sex development (DSD). Mature primary Sertoli cells are incapable of proliferating in prolonged *in vitro* cultures and the available Sertoli cell models have several limitations since they derive from mouse or human cancer tissues. We differentiated human fibroblasts (HFs)-derived induced pluripotent stem cells into Sertoli-like cells (SLC) and, in order to characterize this new Sertoli cell model, we performed gene expression analyses by NextGeneration Sequencing techniques. This approach revealed that our putative SLC have reduced expression of pluripotency markers and expressed Sertoli cell markers such as SRY-Related HMG-Box 9 (SOX9), vimentin (VIM), and claudin-11 (CLDN-11). More in detail, the transcriptional profile analysis suggested that these cells are in an early stage of Sertoli cells maturation. Harnessing the power of induced pluripotent stem cells, we were able to generate SLC that show genetic and functional similarities to human Sertoli cells (HSerCs). SLC could become an excellent source of patient-specific Sertoli cells that could be of paramount benefit for both basic research and personalized medicine in sex development and reproductive medicine.

## Introduction

The normal development of a male individual depends on the development of a functional testis where Sertoli cells (SCs) play a pivotal role. Failure of Sertoli cells function might result in 46,XY disorders of sex development (DSD) with total or partial sex reversion or abnormal production of spermatozoa during adulthood (Michala et al., 2008). One of the main obstacles in the management of DSD is the lack of a correct understanding of their pathophysiology as the causes of DSD are highly heterogeneous, with more than 60 genes reported by now to be associated with sex determination and differentiation (Mendonca et al., 2009; Ono and Harley, 2013; Eid and Biason-Lauber, 2016). The availability of specific cell models for the diverse variants might help to better understand the mechanisms involved in human sex determination and development and to implement more efficient treatments for DSD patients.

SCs are central players in the determination of the testis function as an endocrine (testosterone-secreting) and reproductive (germ cells-producing) organ. SCs cells are the first cell type to differentiate in the bipotential fetal male gonad, in turn enabling testicular cord formation and controlling the differentiation and function of Leydig cells (Racine et al., 1998). Leydig cells, for their part, secrete testosterone and therefore play important roles in downstream masculinization events (Barsoum and Yao, 2010).

SCs proliferate during two periods of life, in neonatal life and in the peripubertal period. In the XY gonad, SCs proliferation is initiated by the expression of the sex-determining region Y chromosome gene (*SRY*), within the genital ridge (at 12.5 days post-coitum in the mouse, Gubbay et al., 1990; and 6–9 weeks after conception in humans, Bendsen et al., 2003). *SRY* lead a series of signaling events and developmental processes that ensure normal testis development. Expression of SRY-related HMG-Box 9 (*SOX9*), a target of *SRY*, in gonadal somatic cells is sufficient to induce the differentiation of SCs even in the absence of *SRY* (Sekido et al., 2004). One morphologically distinct event in testis development is the aggregation of the SCs and primordial germ cells to form testicular cords. As the cords develop, SCs attract endothelial cells from the coelomic epithelium and from the mesonephros. Endothelial cells migrate into the gonad and contribute to the characteristic male pattern of vasculature (Combes et al., 2009; Cool et al., 2011). After that, SCs become quiescent for a variable period depending on the species (Sharpe et al., 2003), showing a second wave of proliferation due to increased gonadotropins at puberty (Cortes et al., 1987; Tarulli et al., 2012). The SCs maturation involves changes in gene transcription and protein expression together with the cessation of proliferation and the establishment of the blood-testis barrier (BTB) (Table 1). Mature SCs are then capable of sustaining spermatogenesis (Lucas et al., 2014). This dual role of SCs highlights their importance in two critical events separated by time and function: the sexual determination and spermatogenesis.

**Table 1:** List of genes from expressed in the different stages of differentiation and maturation of SCs based on literature search.

Symbol	Description	Cell stage	Species	Reference
SOX2	*SRY-Box 2*	PL	Human	Herreros-Villanueva et al., 2013
POU5F1	*POU domain class 5, transcription factor 1*	PL	Mouse	Avilion et al., 2003
DPPA2	*Developmental Pluripotency Associated 2*	PL	Human, Mouse	Kent et al., 1996; Madan et al., 2009; Friedmann-Morvinski and Verma, 2014
DPPA4	*Developmental Pluripotency Associated 4*	PL	Human, Mouse	Kent et al., 1996; Madan et al., 2009; Friedmann-Morvinski and Verma, 2014
NANOG	*Homeobox Transcription Factor Nanog*	PL	Mouse	Choi et al., 2012
NR0B1	*Nuclear Receptor Subfamily 0 Group B Member 1*	PRE	Human, Mouse	Hanley et al., 2000; Stevant et al., 2018
SRY	*Sex Determining Region Y*	PRE	Human, Mouse	Hacker et al., 1995; Hanley et al., 2000; Boyer et al., 2006; Wilhelm et al., 2007; Stevant et al., 2018
AMH	*Anti-Mullerian Hormone*	ES	Human	Franke et al., 2004; Hero et al., 2012
FGF9	*Fibroblast Growth Factor 9*	ES	Mouse	Hanley et al., 2000; Kim et al., 2006; Stevant et al., 2018
CYP26B1	*Cytochrome P450 Retinoid Metabolizing Protein*	ES	Mouse	McClelland et al., 2011
PTGDS	*Prostaglandin D2 Synthase*	ES	Human, Mouse	Malki et al., 2005; Moniot et al., 2009; O’Hara et al., 2015; Stevant et al., 2018
SRC	*Proto-Oncogene Tyrosine-Protein Kinase Src*	ES	Human	Rajpert-De Meyts et al., 1999
KRT18	*Keratin 18*	ES	Human	Franke et al., 2004; Nicholls et al., 2012
DHH	*Desert Hedgehog*	ES	Mouse	Stevant et al., 2018
HSD17B3	*Hydroxysteroid 17-Beta Dehydrogenase 3*	ES	Mouse	Stevant et al., 2018
INHBB	*Inhibin Beta B Subunit*	ES	Human	Hero et al., 2012
CLDN11	*Claudin 11*	LS	Human, Mouse	Baccetti et al., 1998; Moroi et al., 1998; Hellani et al., 2000; McCabe et al., 2016
AR	*Androgen Receptor*	LS	Human	Berensztein et al., 2006
FSHR	*Follicle Stimulating Hormone Receptor*	LS	Human	Baccetti et al., 1998
SOX9	*SRY-Related HMG-Box 9*	AS	Human, Mouse	Kent et al., 1996; Barbara et al., 1998; Hanley et al., 2000; Stevant et al., 2018
VIM	*Vimentin*	AS	Human	Franke et al., 2004
WT1	*Wilms Tumor 1*	AS	Mouse	Stevant et al., 2018
GATA4	*GATA Binding Protein 4*	AS	Human	Ketola et al., 2000

*PL, Pluripotent cell; PRE, Pre-Sertoli cell; ES, Early stage Seroli cell; LS, Late stage Sertoli cells; AS, All Sertoli cells.*

One major hurdle in studying SCs is that mature SCs are mitotically inactive, and primary immature SCs lose their unique characteristics during prolonged culturing. Therefore, finding an alternative source of these cells independent of donor testis cells is of utmost interest both for basic research and clinical applications.

Two main cell models were developed in this respect, the TM4 and the NT2d1 cells. The mouse TM4 cell line was the first Sertoli cell line established by passing cultures of immature mouse SCs over many generations to generate an immortal line (Mather, 1980). Nevertheless, murine models have demonstrated to differ from humans in several genetics regulations involved in sex determination and development such as *SRY* regulation and MAPKs pathways (McClelland et al., 2011; Warr et al., 2011; Larney et al., 2014). NT2d1 cells, in contrast, are human pluripotent clonal cells derived from a testicular tumor (Andrews et al., 1984) and have been shown to express the majority of genes involved in mammalian sex determination (Barbara et al., 1998). Due to their origin, these cell models are not ideal and have limitations if compared with human functional SCs (McClelland et al., 2011; Warr et al., 2011; Larney et al., 2014). Recently, primary human Sertoli cells (HSerCs) have been considered for human SCs studies (Chui et al., 2011; Jesus et al., 2016). Primary HSerCs are supposed to be a reliable model of SCs but they are unable to reproduce the phenotype of DSD patient’s SCs, their collection is difficult and painful, and their expansion in culture is very limited. Thus, an easy to obtain, patient-derived SC model is necessary in order to study the patient-specific Sertoli cell functionality.

Human induced-pluripotent stem cells (iPSCs) have been developed as a powerful cell source for applications in regenerative medicine and drug discovery, primarily based on their extensive similarities to their human embryonic stem cell counterparts and shared properties of self-renewal and multilineage differentiation capabilities (Buchholz et al., 2009; Burridge et al., 2012). iPSCs can be derived from somatic cells via ectopic expression of transcription factors first identified by Yamanaka and co-workers (Takahashi and Yamanaka, 2006; Yu et al., 2007). In our quest to develop an *in vitro* human SC model, we set to use iPSCs. To this end, we generated iPSCs from terminally differentiated human fibroblasts (HFs) and guided their differentiation into Sertoli-like cells (SLC) by the use of the growth factors involved in Sertoli cells differentiation BMP4, basic (b)FGF, prostaglandin D2 (PGD2), fibroblast growth factor 9 (FGF9) and activin A. The new SLCs were characterized by using NGS analysis and compared with the currently available models. Due to the reproducibility of the process and the similarities observed with immature SCs, SLCs become an exceptional source to build patient-specific SC models to study the different DSDs.

## Materials and Methods

### Cell Lines and Animals

Human foreskin fibroblast (HFFn, PC501 A-HFF, System Biosciences Mountain View, CA, United States) were cultured in DMEM medium supplemented with 10% FBS and 1% Pen/Strep according to the manufacturer instructions.

NT2d1 embryonal carcinoma cells (NTERA-2 cl.D1, American Type Culture Collection, Manassas, VA, United States) were grown in DMEM supplemented with 10% FBS and 1% Pen/Strep. NT2d1 RNA was sequenced and used as a Sertoli cell model. Additionally, NT2d1 cells were also used as a feeder layer for colonies differentiation by treating them with Mitomycin C (MMC) to inhibit proliferation. Primary human Sertoli cells (HSerC, ScienCell Research Laboratories, Carlsbad, CA, United States) were cultured in poly-L-lysine coated flasks with SerCM medium (ScienCell Research Laboratories, Carlsbad, CA, United States) supplemented with 1% SerCGS (ScienCell Research Laboratories, Carlsbad, CA, United States), 5%FBS and 1% Pen/Strep according to the manufacturer instructions. HEK293T cells (HCL4517, GE Healthcare, Buckinghamshire, United Kingdom) were cultured in DMEM supplemented with 10% FBS and 1% Pen/Strep. SNL Feeder cells (CBA-136, Cell Biolab, San Diego, CA, United States) were cultured in DMEM supplemented with 10% FBS, 1% Pen/Strep and 0.1 mM MEM Non-essential Amino Acids (NEAA), when a confluence of about 96% was reached, SNL feeder cells were treated with MMC and seeded in Matrigel coated plates as a feeder layer for HFs de-differentiation.

NOD-SCID common gamma2- deficient (NSG) female mice (University of Lausanne, Epalinges, Switzerland) were a kind gift from Prof. Curzio Ruegg (University of Fribourg, Switzerland). Eight weeks old animals were used for all *in vivo* teratoma formation as proof of pluripotency. Animal experiments were carried out according to national ethical guidelines and were approved by the veterinary service of Canton Fribourg (2014_58_FR).

### Reprogramming of HFs Into iPSCs

HFs reprogramming was performed by lentivirus transduction based on Yamanaka and Thomson factors (Takahashi and Yamanaka, 2006; Yu et al., 2007). Lentivirus production was carried out using SIN4-EF2-O2S (Addgene plasmid # 21162) encoding POU domain class 5, transcription factor 1 (*POU5F1) (also known as OCT4)* and Sex Determining Region Y-Box 2 (*SOX2)*, SIN4-EF2-N2L (Addgene plasmid # 21163) encoding Homeobox Transcription Factor Nanog (*NANOG)* and Lin-28 Homolog *(LIN28)*; and SIN4-CMV-K2M (Addgene # 21164) encoding *KLF4* and *C-MYC*. As packaging plasmids we used MD2.G (Addgene plasmid # 12259), CMV (Addgene plasmid # 8455), and PAX2 (Addgene plasmid # 12260). We included a positive transfection control by adding CS-CG plasmid (Addgene plasmid # 12154) encoding *GFP* (data not shown). All plasmids were provided by Addgene (Cambridge, MA, United States).

**FIGURE 1:**
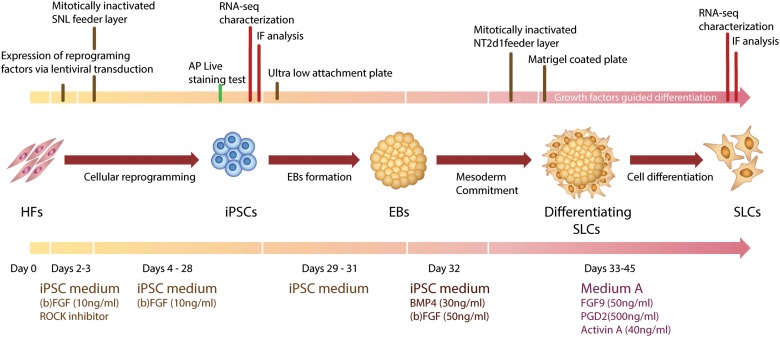
Graphic representation of Human Fibroblasts (HFs) cell reprogramming and iPSCs differentiation toward SLCs. AP, alkaline phosphatase; EBs, embrioid bodies; FGF9, fibroblast growth factor 9; IF, immunofluorescence; iPSCs, induced pluripotent stem cells; PGD2, prostaglandin 2; RNA-seq, next generation RNA sequencing; SLCs, Sertoli-like cells; SNL, SNL feeder cells.

Infective particles were produced by transfection of HEK293T cells. Lentiviruses were collected and HFs were infected 24 and 48 h after transfection. A day after the last transduction round, HFs were seeded over a feeder layer of mitotically inactivated SNL cells in gelatin pre-coated wells (Thermo Fisher Scientific, Waltham, MA, United States) and medium was changed to iPSC medium composed of F12 medium (Sigma-Aldrich, St. Louis, MO, United States) supplemented with 10% Knockout serum replacement (KSR, Gibco Laboratories, Gaithersburg, MD, United States) to avoid any genotoxic effect (Rodríguez-Pizà et al., 2010; Prakash Bangalore et al., 2017), 5% no essential amino acids (NEAA, Gibco Laboratories, Gaithersburg, MD, United States) 1% 2-Mercaptoethanol, 5% penicillin/streptomycin and bFGF (10 ng/ml). Medium was changed every day until small iPSC-like colonies appeared. To check pluripotency *in vivo*, colonies were stained with Alkaline Phosphatase Live (A 14353, Life Technologies, Carlsbad, CA, United States) (Figure 1).

### Teratoma Formation

Four different colonies were grown to confluency in 10 cm dishes, harvested, spinned down, re-suspended in 40 μl DMEM/F12, mixed with Matrigel (1:5) and kept on ice. Twelve female NSG Mice (4–6 weeks old) were injected sub-cutaneously with the cells/Matrigel mix. For each colony, three fractions were prepared and injected in three different mice. The four colonies developed visible tumors in the mice, tumors were then resected and fixed in paraformaldehyde (PFA).

### Differentiation of iPSCs Into SLCs

iPSC colonies were picked by 10 mg/ml collagenase IV treatment. Only colonies with no spontaneous differentiation spots were selected and seeded in poly 2-hydroxyethyl methacrylate (polyHEMA, Sigma-Aldrich, St. Louis, MO, United States) coated ultralow attachment plates with iPSC Medium without growth factors to form embryoid bodies (EBs) during 48–72 h. To induce mesoderm commitment in the EBs, the iPSC medium was supplemented with the specific growth factors bFGF (50 ng/ml) and BMP4 (30 ng/ml) for 24 h. EBs were then transferred to a MMC-treated NT2d1 feeder layer. Medium A containing FGF9 (50 ng/ml) (Sigma-Aldrich, St. Louis, MO, United States), PGD2 (1:2000) (Santa Cruz Biotechnolopy Inc., Santa Cruz, CA, United States) and activin A (40 ng/ml) (Gibco Laboratories, Gaithersburg, MD, United States) was changed daily until EBs spread flat into colony-like structures. Colonies were re-suspended in medium A and seeded on Matrigel-coated wells. The colonies reached confluence after a maximum of 12 days, when they were collected for _q_RT-PCR analysis.

### _q_RT-PCR Analysis

qRT-PCR analysis was performed as described previously (Eid et al., 2015). Briefly, extracted total RNA by Qiagen RNAeasy plus (Qiagen, Hilden, Germany) was reverse-transcribed using Omniscript reverse-transcriptase (Qiagen, Hilden, Germany) according to the manufacturer instructions. All experiments were performed on an ABI StepOnePlus Real-Time PCR (Thermo Fisher Scientific, Waltham, MA, United States) and the PCR products were quantified fluorometrically using the KAPA SYBR FAST master mix (Roche, Basel, Switzerland). To normalize the data, the mRNA level of cyclophilin was used.

### RNA-Seq Analysis

RNA-sequencing data collection and analysis were carried out by the Functional Genomic Center Zurich (Zurich, Switzerland). Total RNA samples from NT2d1 triplicates, TM4 cell triplicates, SLCs and HSerCs were analyzed by RNA-Sequencing on a HiSeq 2500 Sequencer (Illumina, San Diego, CA, United States). The reads were screened with FastQ Screen (Babraham Bioinformatics) for possible contamination and a quality control has been performed with FastQC (Babraham Bioinformatics). RSEM (Dewey Lab) was used to quantify the gene expression level and the differential expression between samples. The corresponding false discovery rate (FDR), was calculated by EdgeR (Bioconductor). The FDR is defined as the probability of a false-positive discovery, taking into account the total number of null hypotheses tested over the whole experiment. All differential expressions with a FDR below 0.05 were defined as significant. STAR (Spliced Transcripts Alignment to a Reference) was used to map the RNA reads to the reference sequence. Sequencing data are available at ENA repository under the ID PRJEB22617.

### Immunofluorescence

Immunofluorescence was carried out as previously described (Eid et al., 2010) Briefly, iPSC colonies, Matrigel-coated and ECM 3D gel cells were fixed in 4% paraformaldehyde in PBS and permeabilized with 0.2% Triton X-100 in PBS. For pluripotency assessment in iPSC colonies, cells were blocked in 3% BSA/PBS for 1 h, washed 3 times in PBS and then incubated with primary antibodies against OCT4 (1:200, BD Bioscience, San Jose, CA, United States Cat# 611202), SOX2 (1:100, DSHB, Iowa City, IA, United States Cat#) TRA-1-81 (1:200) SSEA1 (MC-480), SSEA3 (MC-631), and SSEA4 (MC-813-70) (1:3, deposited to the DSHB by Solter, D., Knowles, B.B) overnight at 4°C. For Sertoli-like characterization, primary antibodies against SOX9 (Cat# MA5-17177, Thermo Fisher Scientific, Rockford, IL, United States), FOXl2 (Cat# LS-B12865, LifeSpan Bioscience), CLDN11 (Cat# ab53041, Abcam, Cambridge, United Kingdom) were diluted 1:500 and incubated overnight at 4°C.

Secondary antibodies Alexa Fluor 488 conjugated goat anti-rabbit (Cat# A-11008), Alexa Fluor 594 conjugated goat anti-mouse (Cat# A-11005) and Alexa Fluor 488 bovine anti-goat (Cat# A-11055, Thermo Fisher Scientific, Rockford, IL, United States) were diluted 1:1000 and incubated protected from light for 1 h at room temperature. Mounting medium containing DAPI (Vectashield, Burlingam, CA, United States) was added and incubated for 20 min at room temperature before samples were examined under immunofluorescence with a CKX41 inverted microscope (Olympus Corporation, Tokyo, Japan).

### Anti-Mullerian Hormone (AMH) Quantification

Cells were incubated with equal amount of F-12 medium + 10% Knockout serum replacement + 1% Pen/strep for a period of 2 h. After incubation, the medium was collected and centrifuged at 1000x rpm for 20 min to discard cell debris. Remaining cells were lysed in a cocktail of NP40 (AMRESCO LLC, Solon, OH, United States), 1 mM PMSF (Thermo Fisher Scientific, Waltham, MA, United States) and a cOmplete ULTRA protease inhibitor tablet (Roche, Basel, Switzerland). The cells lysates were used for total protein quantification by Bradford assay (Bio-Rad Laboratories, CA, United States). Secreted AMH protein was quantified by AMH ELISA kits (Cloud-Clone Corp, TX, United States). Results were analyzed using an Infinite microplate reader (Tecan Trading AG, Switzerland). Values were standardized by the amount of total protein in the lysates.

### Statistics

Quantitative variables are described as mean ± SD. A *P* < 0.05 was considered statistically significant. Unpaired t-tests were performed using GraphPad Prism Software (La Jolla, CA, United States).

## Results

### HFs Are Reprogrammed to iPSCs

**FIGURE 2:**
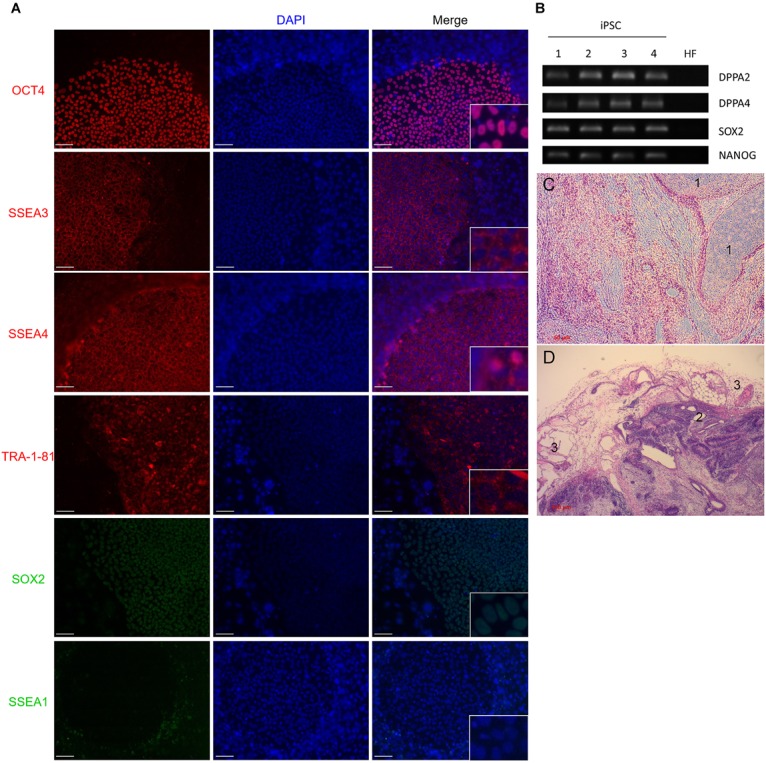
**(A)** Immunofluorescence staining of iPSC colonies. Zoom-in details in right-down squares. Cells were stained with antibodies against OCT4, TRA-1-81, SSEA3, SSEA4, or SSEA1 and SOX2. Mounting medium contains DAPI stain. Pluripotency markers OCT4, TRA-1-81, and SOX2 positively marked the nuclei of cells. SSEA3 and SSEA4 were localized in the cytoplasmic area of colonies. SSEA1 expression was absent in colony nuclei but observed in the feeder cells. **(B)** RT-PCR analysis of pluripotential colonies for the genes DPPA2, DPPA4, ESG1, SOX2, and NANOG. Primers used for SOX2 and NANOG specifically detect transcripts from the endogenous genes, but not from the lentiviral transgenes. Four (1–4) different colonies are shown. HF, terminally differentiated fibroblasts; iPSC, induced pluripotent stem cells. **(C,D)** Teratoma formation analysis. Histology sections of teratoma stained with Hematoxylin & eosin. 1: mesoderm, 2: endoderm, 3: ectoderm. Scale bars 50 μm for **(A,C)**, and 100 μm for **(D)**.

Induction of iPSCs by lentivirus transduction was performed by following a reprogramming protocol as previously described (Yu et al., 2007). Approximately 3 weeks post-transduction we observed defined colonies that were flat and resembled iPSC morphology: small, tightly packed cells with a high nucleus/cytoplasm ratio (Kato et al., 2016). *In vivo* staining with alkaline phosphatase (AP) live stain identified the colonies as potential iPSCs (data not shown). Molecular and functional characterizations were performed to confirm the pluripotency of the colonies. The pluripotent cell markers POU5F1, SSEA3, SSEA4, TRA-1-81, and SOX2 were markedly expressed in our colonies whereas SSEA1, a marker of differentiation in human stem cells could not be detected (Figures 2A,B). This demonstrated that HFs were efficiently reprogrammed into iPSCs colonies.

### iPSC Colonies Are Capable of Generating the Three Different Germ Layers

In order to assess if the generated iPSCs are able to differentiate into the three germ layers, iPSCs were injected into SCID mice that developed tumors 6 weeks post-injection. Tumors were resected and fixed, and slide sections were examined by microscopy for the formation of the three germ layers (teratoma). Microscopic examination of the formed tumors showed that the induced pluripotent stem cells we generated possess the ability to differentiate into the three germ layers (Figures 2C,D).

### After Differentiation Guidance From iPSCs, SLCs Show a High RNA Expression of Early Stage Sertoli Markers When Compared to HSerCs and NT2d1 Cells

**FIGURE 3:**
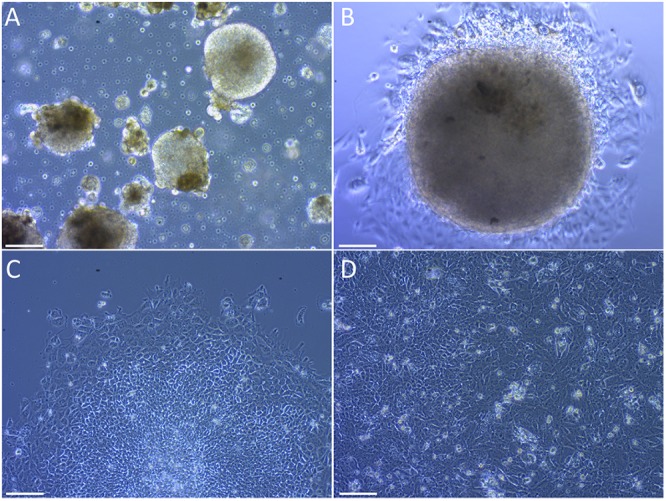
Differentiation of embryoid bodies to Sertoli-like colonies. **(A)** Embryoid bodies growing in suspension. **(B)** Embryoid body attached to the plate bottom after been passed to an NT2d1 feeder layer. **(C)** Colony in differentiation, the undifferentiated center can be appreciated while cells around the borders have already changed their morphology. **(D)** Established SLC colonies. Cells show an elongated shape similar to primary Sertoli cells. Scale bar 100 μm for **(A,C,D)**, and 50 μm for **(B)**.

The formation of embryoid bodies (EBs) is the main step in the embryonic stem cells differentiation (Doetschman et al., 1985). Colonies were successfully induced to form EBs by culturing them in poly-HEMA-coated plates to prevent cell attachment (Figure 3A). A second critical step is the mesoderm commitment, where mesoderm layer is formed via epithelial-to-mesenchymal transition (EMT) (Hay, 1995). iPSCs-derived EBs were induced to mesoderm commitment by culturing them in medium supplemented with BMP4 and bFGF. The differentiation process was guided by a mixture of extracellular signaling molecules that are known to induce Sertoli cell formation in humans. These include FGF9, PGD2, BMP4 and (b)FGF (Pellegrini et al., 2003; Schmahl et al., 2004; Moniot et al., 2009). During the differentiation events, cells in the periphery of the colonies changed their morphology to a multipolar, elongated shape (Figures 3B,C). After 12 days in culture the colonies reached confluency (Figure 3D).

**FIGURE 4:**
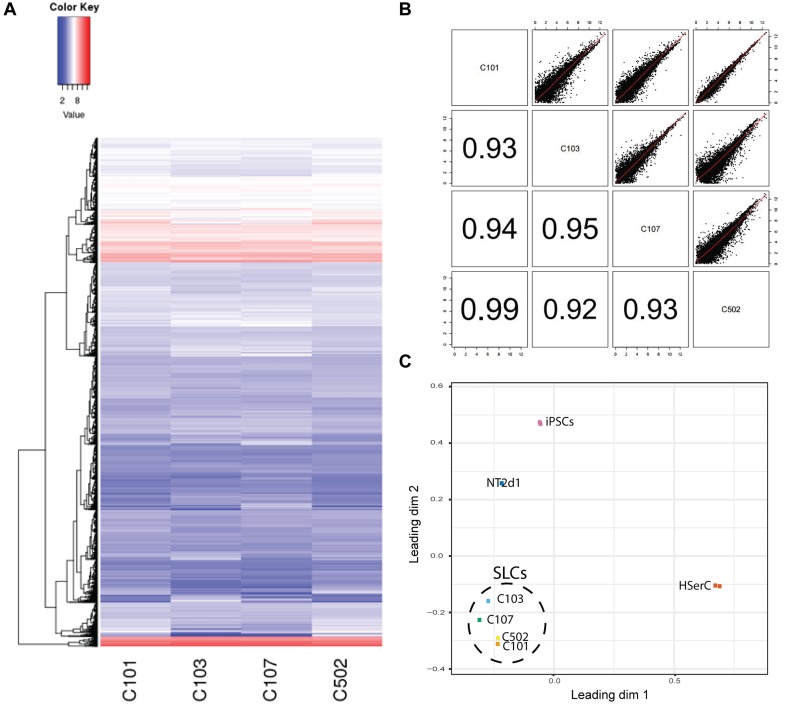
Colonies classification based on gene expression. **(A)** Transcriptome heatmap of genes expressed in the four colonies analyzed (C101, C103, C107, C502). Red color means higher expression and blue color means lower expression than the average. Expression level represented as Fragments per Kilobase Million (FPKM). **(B)** Colony-to-Colony correlation based on gene expression. Upper half of the chart represents scatter plot of all genes analyzed. Lower part represents the correspondent *R*^2^. An *R*^2^> 0.8 was considered significant. **(C)** Principal component analysis.

In order to characterize our SLCs and compare them with the available Sertoli cells models, we performed RNA-sequencing analysis of SLCs derived from four colonies as well as NT2d1, HSerCs, and iPSCs. The transcriptome analysis of 44949 genes showed that RNA expression pattern was similar among SLCs colonies and no significant differences were observed, suggesting that the differentiation protocol is reproducible, the correlations among each other were significantly high in all cases (*R*^2^> 0.90) (Figures 4A,B). When compared to the other cell lines analyzed, SLCs clearly differed from HSerCs, NT2d1 cells or iPSCs (Figure 4C).

**FIGURE 5:**
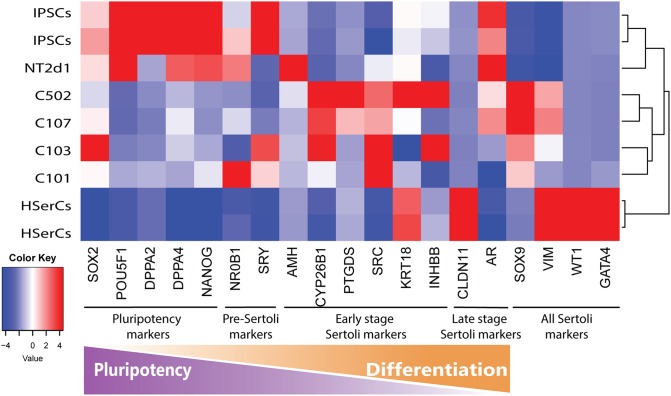
Analysis of genes markers of SCs stages. Transcriptome heatmap of relative gene expression between Sertoli cell models.RNA expression pattern for NT2d1, HSerCs and four SLC colonies. Red color means high expression, blue color means low expression when compared to the mean expression level. *n* = 4 for NT2d1 and SLCs, *n* = 2 for HSerCs and iPSCs.

**FIGURE 6:**
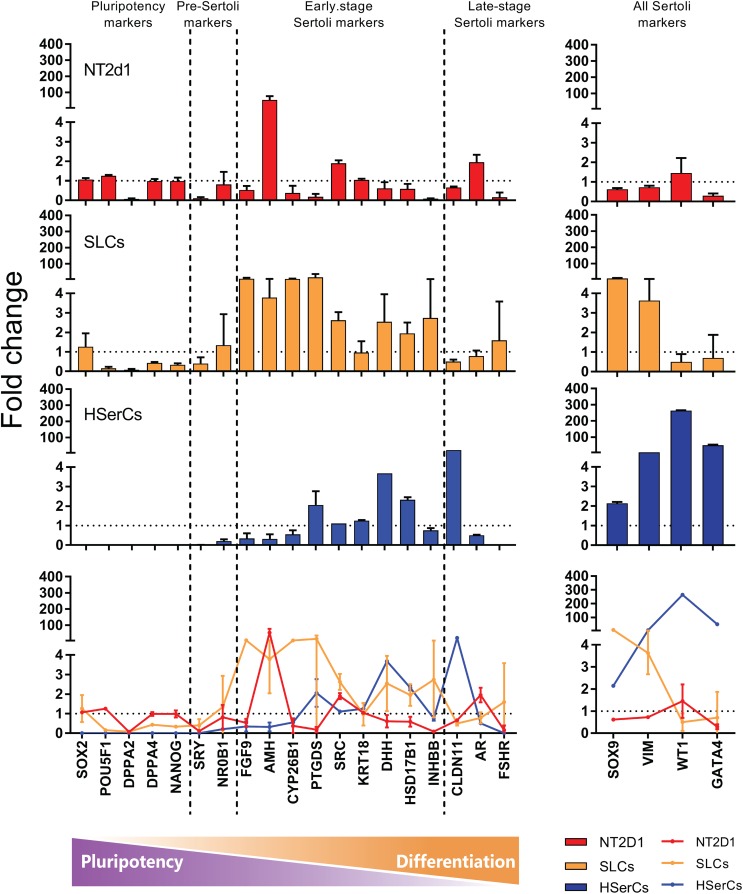
RNA seq expression analysis of genes involved in the Sertoli cell differentiation. Comparison between NT2d1 cells (red), SLCs (green), and HSerCs (blue). All values are relative to iPSCs expresion levels (dotted horizontal line). Additionaly to the expression of the general SC markes *SOX9* and *VIM*, SLC showed an elevated expresion of genes corresponding to early stages of differentiation and maturation of SCs (*FGF9, AMH, CYP26B1, PTGDS, SRC, DHH, HSD17B3*, and *INHBB*). *n* = 4 for NT2d1 and SLCs, *n* = 2 for HSerCs and iPSCs. Colored bars and lines are mean ± SD. HSerCs, human Sertoli cells; NT2d1, NT2d1 cells; SLCs, Sertoli like cells.

Based on literature search from human and rodent sex development studies (Hacker et al., 1995; Kent et al., 1996; Baccetti et al., 1998; Hanley et al., 2000; Hellani et al., 2000; Ketola et al., 2000; Franke et al., 2004; Sekido et al., 2004; Berensztein et al., 2006; Boyer et al., 2006; Kim et al., 2006; Maldonado-Saldivia et al., 2007; Moniot et al., 2009; Choi et al., 2012; Hero et al., 2012; Jameson et al., 2012; Nicholls et al., 2012; Herreros-Villanueva et al., 2013; Larney et al., 2014; McCabe et al., 2016; Stevant et al., 2018), we selected a battery of 23 genes involved in different stages of SCs differentiation and maturation and analyzed their relative expression to iPSCS values for SLCs, NT2d1 and HSerCs (Table 1 and Figures 5, 6).

We analyzed the gene expression pattern of SLCs in comparison with HSerCs (Figure 5). Of the pluripotency markers tested (*SOX2, POU5F1, DPPA2, DPPA4*, and *NANOG*), we observed that *DPPA2, DPPA4*, *POU5F1*, and *NANOG* were less expressed in SLCs than iPSCs. All the gene markers of all the steps of Sertoli cell maturation (*SOX9*, *VIM*, *WT1*, and *GATA4)* where highly expressed in HSerCs, as expected. However, SLCs only shows high expression of *SOX9* and *VIM* but not *WT1* or *GATA4*. Interestingly, when we looked at specific markers of immature Sertoli cells (*SRC, FGF9, AMH, CYP26B1, PTGDS, KRT18, DHH, HSD27B3*, and *INHBB*), SLCs showed elevated expression of all of them but KRT18, this expression was remarkable in the case of *FGF9, AMH, CYP26B1, and PTGDS*. In contrast, SLCs showed a reduced expression of gene markers characteristic for late stages of maturation (*CLDN11, AR*, and *FSHR*) when compared with HSerCs, this was especially clear for *CLDN11*, a critical player in the formation of tight junctions (TJs) whose maximum expression values correspond to the maturation of Sertoli cell (Stammler et al., 2016). These data were validated by _q_RT-PCR analysis (Supplementary Table S1 and Supplementary Figure S2). Taken together, these results suggested that iPSCs were successfully differentiated into SLCs and are indeed in an early stage of Sertoli cell maturation. Thus, we considered SLCs as an immature/differentiating Sertoli cell model, potentially very useful to study developmental processes.

### SLCs Express the Sertoli Cell Marker Proteins SOX9, Claudin-11, and AMH

**FIGURE 7:**
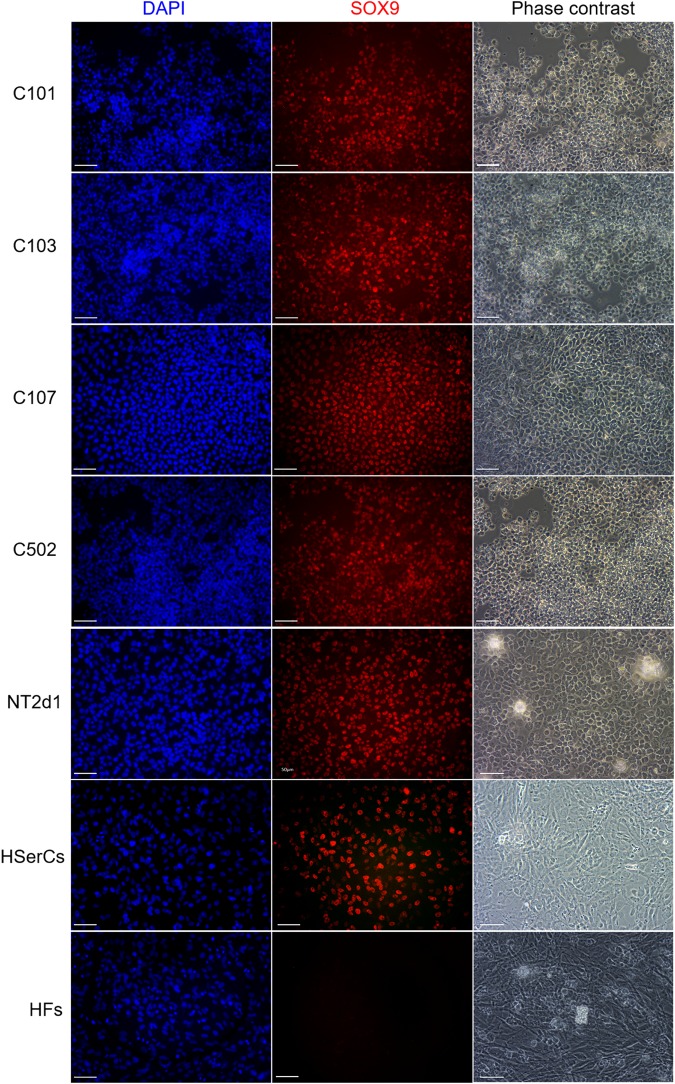
Immunofluorescence staining of SLC colonies with antibodies against SOX9. Mounting medium contains DAPI stain. SOX9 were expressed in SLC colonies. HF, terminally differentiated fibroblasts; HSerCs, human Sertoli cells; NT2, NT2d1 cells; SOX9, SRY (sex determining region Y)-box 9. Scale bars 50 μm.

To test whether protein expression and localization in SLCs are similar to natural Sertoli-cells, we performed immunofluorescence for SOX9 (a Sertoli cell marker) (Kent et al., 1996), and FOXL2 a granulosa cells marker that is not expected to be expressed in a male lineage (a negative control) (Cocquet et al., 2002). SOX9 was not expressed in HFs but was present in the nucleus of all SLCs colonies, NT2d1, and HSerCs (Figure 7). We also observed that SOX9 was consistently expressed in SLCs even after freeze/thawing cycles, which suggest a high stability of the model (data not shown). FOXL2 was undetectable in all the colonies analyzed as well as the HSerCs and HFs, but it was detected in KGN and NT2d1 cells at low levels (Supplementary Figure S3).

**FIGURE 8:**
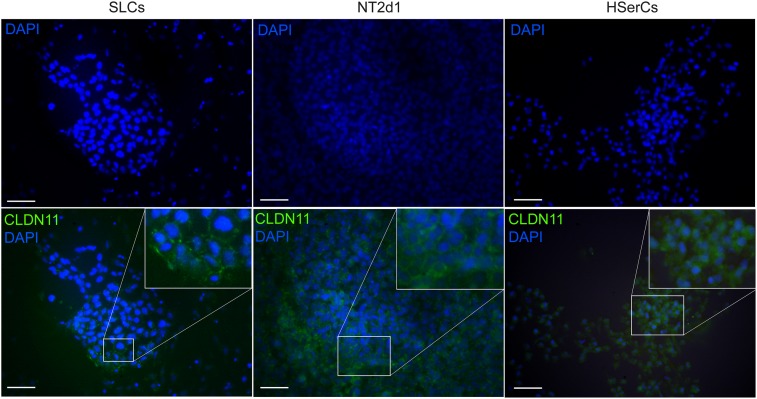
Immunofluorescence staining of SLC colonies with antibodies against CLDN11. Mounting medium contains DAPI stain. Claudin-11 was expressed in SLCs as in NT2d1 and HSerCs. Upper right squares show a magnification detail of the original pictures. CLDN11, claudin-1; HSerCs, human Sertoli cells; NT2d1, NT2d1 cells; SLCs, Sertoli like cells. Scale bars 50 μm.

Human Sertoli cells are known to express claudin-11 whose expression is well conserved among different species and seems essential for TJs formation (Stammler et al., 2016). Despite the low expression of CLDN11 gene by SLCs, we analyzed whether those levels were enough to form TJs by using immunofluorescence analysis (Figure 8). Claudin-11 expression was clearly detectable in SLCs colonies when cells interacted with each other in tight groups. Upon comparison to the other cell models, we observed that claudin-11 was distinctly located in the membrane in SLCs and NT2d1 cells but its location was much more diffuse in the case of HSerCs being predominantly expressed in the cytoplasm. The presence and cellular location of Claudin-11 in SLCs suggests that these cells resemble the natural characteristics of HSerCs that are in the process of maturation.

**FIGURE 9:**
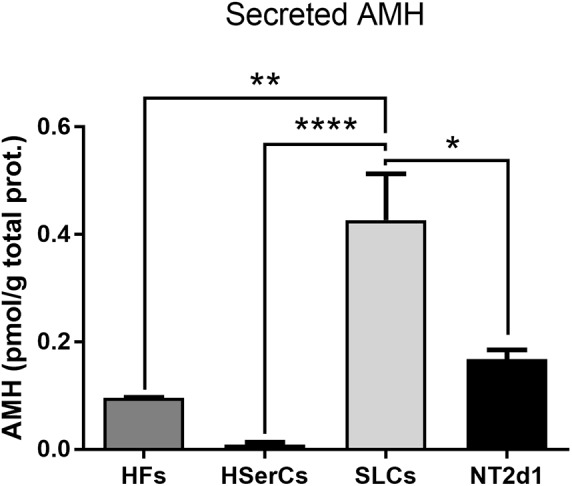
Secreted AMH quantification by ELISA. SLCs secreted AMH levels were significantly higher when compared with AMH secretion by HSerCs or NT2d1 cells. HF: terminally differentiated fibroblasts, HSerCs: primary human Sertoli cells, NT2d1: NT2d1 cells, SLCs: Sertoli like cells. *n* = 4 for SLCs and HSerCs, *n* = 2 for HFs and NT2d1. ^∗^*P* < 0.05, ^∗∗^*P* < 0.01, ^∗∗∗∗^*P* < 0.0001.

AMH is a well-known marker of Sertoli cells. We tested whether the high expression of the *AMH* gene we found in RNA-Seq is reflected in a highly secretion of AMH protein by SLCs. We quantified secreted AMH in the medium of SLCs by ELISA and compared it with AMH secreted by both NT2d1 and HSerCs cell lines as well as HFs. The levels of secreted AMH by SLCs were indeed significantly higher than the AMH secretion of HSerCs or NT2d1 cells (Figure 9).

## Discussion

SCs drive the development of the bipotential gonad toward the male pathway and control proper testis function. The small amount of cells collected in a biopsy together with the impossibility of its prolonged culture prompted the search for alternative cell sources to build a reliable model. Previous cell models were obtained from mouse Sertoli cells (TM4 cell line) or derived from human pluripotent carcinoma tissue (NT2d1 cell line). Mouse cell models like TM4 have shown differences with human SCs in proliferation timing and protein expression. Even when the use of commercially available HSerCs may help to establish a model for healthy adult human SCs, they cannot be used to mimic the particularities of the developing gonad and identify mechanisms of disease in different developmental defects like DSDs. Now, thanks to the power of novel cell reprogramming technologies, we are able to reverse the cellular faith of many different cell types to a pluripotent state. These induced pluripotent cells have the ability to develop into almost every cell type. We hypothesized that using this powerful tool patient’s cells could be reprogrammed and re-differentiated into SLCs, which may become a highly accurate model for the study of SCs in DSD patients.

Human fibroblasts represent a suitable start-point for reprograming methodology since they are terminally differentiated cells that have no resemblance to pluripotent characteristics or similarities with functional SCs. They also constitute a reliable source of human cells and are easy cultured *in vitro*. Moreover, mouse fibroblasts already proved to be successfully reprogrammed into embryonic Sertoli cells (eSCs) (Buganim et al., 2012).

iPSC colonies can be re-differentiated into other cell type by forced expression of reprogramming factors via viral transduction or by the addition to the medium of specific growth factors in a very precise timing (Mummery et al., 2012; Yoder, 2015; Gunhanlar et al., 2017; Pandya et al., 2017) and the choice of these specific factors is crucial for successful differentiation. In our case, (b)FGF, BMP4, FGF9, PGD2 and activin A are a very well-known group of growth factors that induce differentiation from embryonic stem cells (Schuldiner et al., 2000). (b)FGF and BMP4 induce mesoendoderm signals of vital importance for the formation of the urogenital system (Lawson et al., 1999; Evseenko et al., 2010) and they stimulate the expression of *SOX9. SOX9* plays a central role in Sertoli cell differentiation and its expression needs to be maintained during the whole process. PGD2 and FGF9 stimulate in an independent way the expression of *SOX9* (Kim et al., 2006; Moniot et al., 2009). Additionally, Activin A is essential for Sertoli cells early differentiation and proliferation (Nicholls et al., 2012). The consistent elevated expression of *SOX9* in our SLCs is in consonance with an effective stimulation by all the administered growth factors.

A comprehensive characterization of the transcriptional landscape of our SCs based on the expression of differentiation stage-specific markers is essential to determine the suitability of the model. The transition from pluripotent cells to actual Sertoli cells implies the reduction of expression of *POU5F1, SOX2*, and *NANOG*, that act as intrinsic determinants of embryonic stem cells (ESCs) identity (Nichols et al., 1998; Avilion et al., 2003; Chambers et al., 2003). *DPPA2* and *DPPA4* were related to the pluripotency network (Tung et al., 2013) and, even when they are not essential for ESCs identity, seem to be critical for early steps of embryogenesis (Madan et al., 2009). The reduction in expression of these “pluripotency” genes in SLCs compared to their expression in iPSCs denotes that SLC model is advanced in the cell differentiation path. This is of vital importance since the loss of those pluripotency markers reduces the risk of spontaneous dedifferentiation (Friedmann-Morvinski and Verma, 2014), making the model more stable.

Male sex determination is triggered by the early expression of *SRY* (Goodfellow and Lovell-Badge, 1993) followed by its stimulation of *SOX9*. a pivotal sex-determining gene and a milestone for checking the differentiation state of SLCs. The clear expression of *SOX9* denotes that our SLCs were stably channeled toward the male determination pathway.

The high expression tendency observed for the markers *PTGDS*, *FGF9, AMH, CYP26B1*, and *IHNBB* (Figure 5 and Supplementary Figure S1), suggested that SLCs colonies are in early stage of maturation. PTGDS and FGF9 are expressed during SCs differentiation shortly after SRY and SOX9 and contributes to SOX9 nuclear translocation (Malki et al., 2005; Kim et al., 2006; Wilhelm et al., 2007). CYP26b1 also acts after SRY is expressed and reinforce the male differentiation pathway by stimulating SOX9 and FGF9 (McClelland et al., 2011). The elevated expression of SRC, which is involved in the binding between SCs and germ cells and in the sperm release (O’Hara and Smith, 2015) together with the observed levels of *INHBB* and VIM suggest maturation, albeit again not complete. AMH is routinely used as a marker for Sertoli cells function during development and its production is known to be triggered by SOX9 (among other factors). In accordance to this, *AMH* expression and secretion was increased in SLCs when compared to HSerCs in parallel to SOX9. It is also known that the reduction of *AMH* expression levels likely reflect SCs maturation (Hero et al., 2012) and matches the fact that SCs maturation involves the loss of expression of fetal markers after puberty (Rajpert-De Meyts et al., 1999). Taken together, these results suggest that our cells are indeed in an intermediate state of differentiation/maturation while HSerCs show a more advanced maturation phenotype. In the gonads, AMH is produced as an inactive pro-protein (proAMH) which is folded in its C-terminus. Pro-AMH is cleaved in a later stage by serine protease into two different fragments linked to each other (McLennan and Pankhurst, 2015). Although speculative, this higher total AMH secretion in SLCs may be explained by post-translational modifications of pro-AMH or its active form, preventing the protein degradation and promoting its accumulation in the medium. Interestingly, NT2d1 cells showed higher *AMH* gene expression than SLCs but significantly lower protein secretion, suggesting that AMH secreted protein is more stable in SLCs than NT2d1 cells.

Another critical feature of mature SCs is the creation and maintenance of the BTB and their relations with the basement membrane, a modified form of extracellular matrix (ECM). Proteins in the ECM were shown to regulate BTB dynamics via the interactions between collagens, proteases and protease inhibitors. It was postulated that ECM regulates Sertoli cell tight-junctions barrier function and spermatogenesis *via* integrins, which in turn transmit signals to regulate junction dynamics (Siu et al., 2003). The BTB is maintained thanks to the tight-junctions between mature SCs, preventing the entrance of molecules to luminal space of the seminiferous tubules. Occludins and claudins are common elements of the TJs in several species but only claudin-11 expression is well conserved among mouse, rat and human, emphasizing its essential role in the BTB structure (Stammler et al., 2016). In rodents, claudin-11 has shown to be regulated by FSH and testosterone and plays a key role in the formation of the SCs TJs, with maximal expression corresponding to the creation of the BTB (Hellani et al., 2000). In humans, claudin-11 presence is almost exclusive of Sertoli cells (and oligodendrocytes in the central nervous system) (Moroi et al., 1998; McCabe et al., 2016). Accordingly, expression levels of CLDN11 were high in HSerC, indicating their mature state. Since the levels of CLND11 in SLCs were lower we tested whether the protein levels were enough to form proper TJs. The presence of claudin-11 in SLCs junctions suggested that our SLC model is indeed in the process of maturation, being able to create cell-to-cell connections in the same way that natural human SCs do. This ability is of great interest to study co-cultures with other testicular cells types like spermatogonia or myoid cells.

## Conclusion

We were able to reprogram and re-differentiate terminally differentiated HFs into SLCs. This new SLC model resembles human SCs in morphology and gene expression. Furthermore, our model showed protein expression of human SCs markers like *SOX9, VIM, SRC* and Claudin-11, making them a reliable model for human SCs. From a more clinical point of view, given the difficulties to recapitulate the human phenotype using classical animal models, these results offer the opportunity to generate patient-specific SCs models that, together with the novel techniques in next generation sequencing, will allow the study of mechanism of disease for single patients and may boost the understanding of the individual complexities and help to refine the aim of treatments for human DSDs. Furthermore, knowledge acquired by studying rare diseases might help clarify the still unknown pathophysiology of more common and complex entities such as male in- and decreasing fertility.

## Author Contributions

DRG, WE, and AB-L conceived and designed the experiments and wrote the paper. DRG and WE performed the experiments, analyzed the data, and contributed reagents, materials, and analysis tools.

## Conflict of Interest Statement

The authors declare that the research was conducted in the absence of any commercial or financial relationships that could be construed as a potential conflict of interest.
