# Supplementary material for: A Human Gonadal Cell Model From Induced Pluripotent Stem Cells
Source: Front Genet. 2018 Oct 24;9:498. doi: 10.3389/fgene.2018.00498 (PMC6207579; doi:10.3389/fgene.2018.00498)
Supplement: Supplementary file 1 [file Data_Sheet_1.PDF]

Supplementary Material

A Human Gonadal Cell Model from Induced Pluripotent Stem Cells.

Daniel Rodríguez Gutiérrez[1,3], Wassim Eid[1,2,3], Anna Biason-Lauber[1*]

[1]Endocrinology division, Section of Medicine, University of Fribourg, Fribourg, Switzerland

[2]Department of Biochemistry, Medical Research Institute, University of Alexandria, Alexandria, Egypt

[3]These authors contributed equally to this work.

*Corresponding Author:

Anna Biason-Lauber, MD, Prof.
Endocrinology Section of Medicine

University of Fribourg
Chemin du museé 05
1700 Fribourg
Switzerland
Phone: +41 26 300 8534
E-mail: (ABL) anna.lauber@unifr.ch

1 Supplementary Tables

Gene	Fordward sequence	Reverse sequence
SOX2	5'-GCTACAGCATGATGCAGGACCA-3'	5'-TCTGCGAGCTGGTCATGGAGTT-3'
OCT4	5'-CCTGAAGCAGAAGAGGATCACC-3'	5'-AAAGCGGCAGATGGTCGTTTGG-3'
DPPA2	5'-GGACTGGTGTCAACAACTCGGT-3'	5'-TCACTGCCTTGCGTTTCCTCGA-3'
DPPA4	5'-CTCCACAGAGAAGTCGAGGGAA-3'	5'-GGTTGTCAGTGTGCTCTGCCTT-3'
NANOG	5'-CTCCAACATCCTGAACCTCAGC-3'	5'-CGTCACACCATTGCTATTCTTCG-3'
NR0B1	5'-CCAAATGCTGGAGTCTGAACATC-3'	5'-CCCACTGGAGTCCCTGAATGTA-3'
SRY	5'-GCTGCAAGGGCTTCTTCAAGCG-3'	5'-GCCACGAGGTCTTCGGTTCAGT-3'
AMH	5'-CGCTGCTTCACACGGATGACC-3'	5'-GGTGGCGACTCCTCGAGTTCC-3'
FGF9	5'-CCAGGAAAGACCACAGCCGATT-3'	5'-CCATACAGCTCCCCCTTCTCAT-3'
CYP26B1	5'-GCACCTCTTTGAGGTCTACCAG-3'	5'-AGGATCTGCCGAGCCTGAATGC-3'
PTGDS	5'-AGCCCACTTCCAGCAGGACAA-3'	5'-CAGACTTGCACATGGACAACGC-3'
SRC	5'-CTGCTTTGGCGAGGTGTGGATG-3'	5'-CCACAGCATACAACTGCACCAG-3'
KRT18	5'-GCTGGAAGATGGCGAGGACTTT-3'	5'-TGGTCTCAGACACCACTTTGCC-3'
DHH	5'-AGGATGAGGAGAACAGTGGAGC-3'	5'-TCAGTCACTCGTAGGCGCACTC-3'
HSD17B3	5'-TTGGAGGTGAAACCTGTGGCTG-3'	5'-CTACCTGACCTTGGTGTTGAGC-3'
INHBB	5'-GAAATCATCAGCTTCGCCGAGAC-3'	5'-GGCAGGAGTTTCAGGTAAAGCC-3'
CLDN11	5'-GGCTGGTGTTTTGCTCATTCTGC-3'	5'-AGCACCAATCCAGCCTGCATAC-3'
AR	5'-ATGGTGAGCAGAGTGCCCTATC-3'	5'-ATGGTCCCTGGCAGTCTCCAAA-3'
FSHR	5'-GGTTTGTCCTCACCAAGC-3'	5'-GCTTGGAGAACACATCTG-3'
SOX9	5'-GGCTACGACTGGACGCTGGT-3'	5'-TGCTGAGCTCGGCGTTGTGC-3'
VIM	5'-AGGCAAAGCAGGAGTCCACTGA-3'	5'-ATCTGGCGTTCCAGGGACTCAT-3'
WT1	5'-CGAGAGCGATAACCACACAACG-3'	5'-GTCTCAGATGCCGACCGTACAA-3'
GATA4	5'-GCGGTGCTTCCAGCAACTCCA-3'	5'-GACATCGCACTGACTGAGAACG-3'
PPIA	5'-GGCAAATGCTGGACCCAACACA-3'	5'-TGCTGGTCTTGCCATTCCTGGA-3'

Table S1: List of primer pairs used for qRT-PCR analysis.

1.1 Supplementary Figures

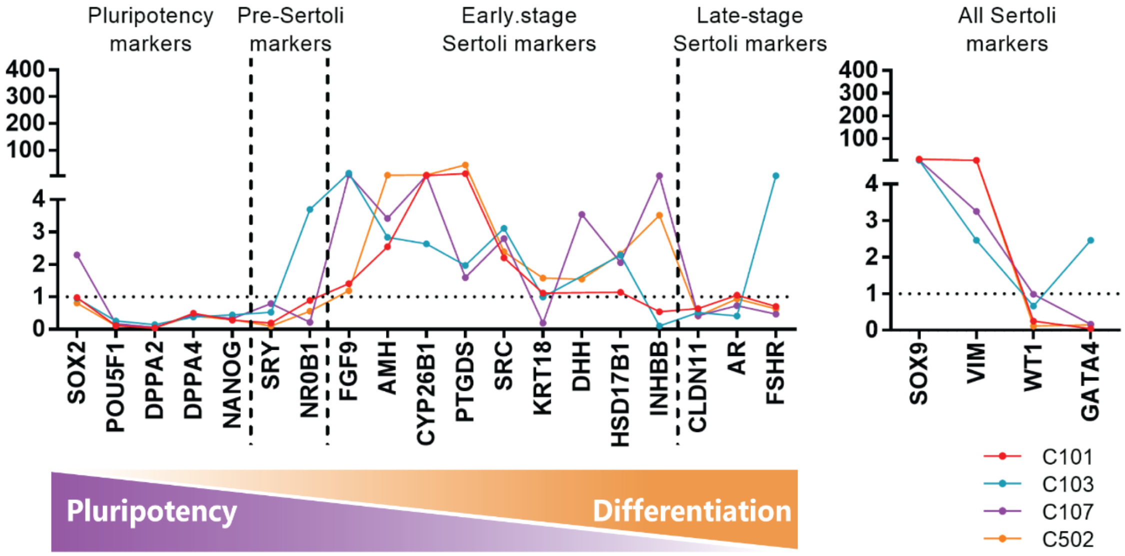

Supplementary figure S1: RNA seq gene expression analysis of SCs marker for single colonies. Comparison between C101 (red), C103 (blue), C107 (purple) and C502 (orange). All values are relative to iPSCs expression levels (dotted horizontal line).

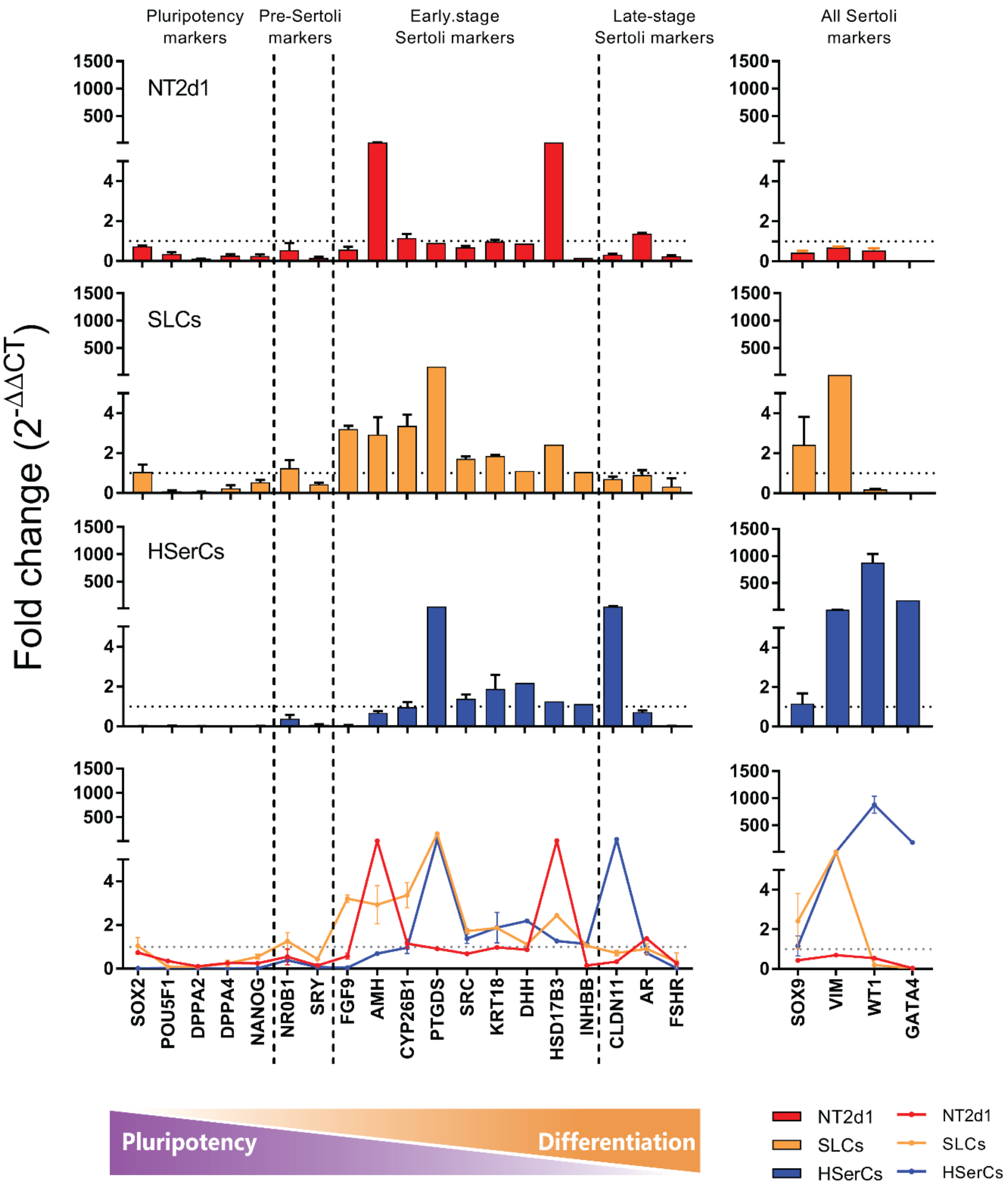

Supplementary figure S2: qRT-PCR analysis of Sertoli-like single colonies. Colored bars and lines are mean ± SD. HSerCs: human Sertoli cells, NT2d1: NT2d1 cells, SLCs: Sertoli like cells. n=4 for NT2d1 and SLCs, n=2 for HSerCs and iPSCs.

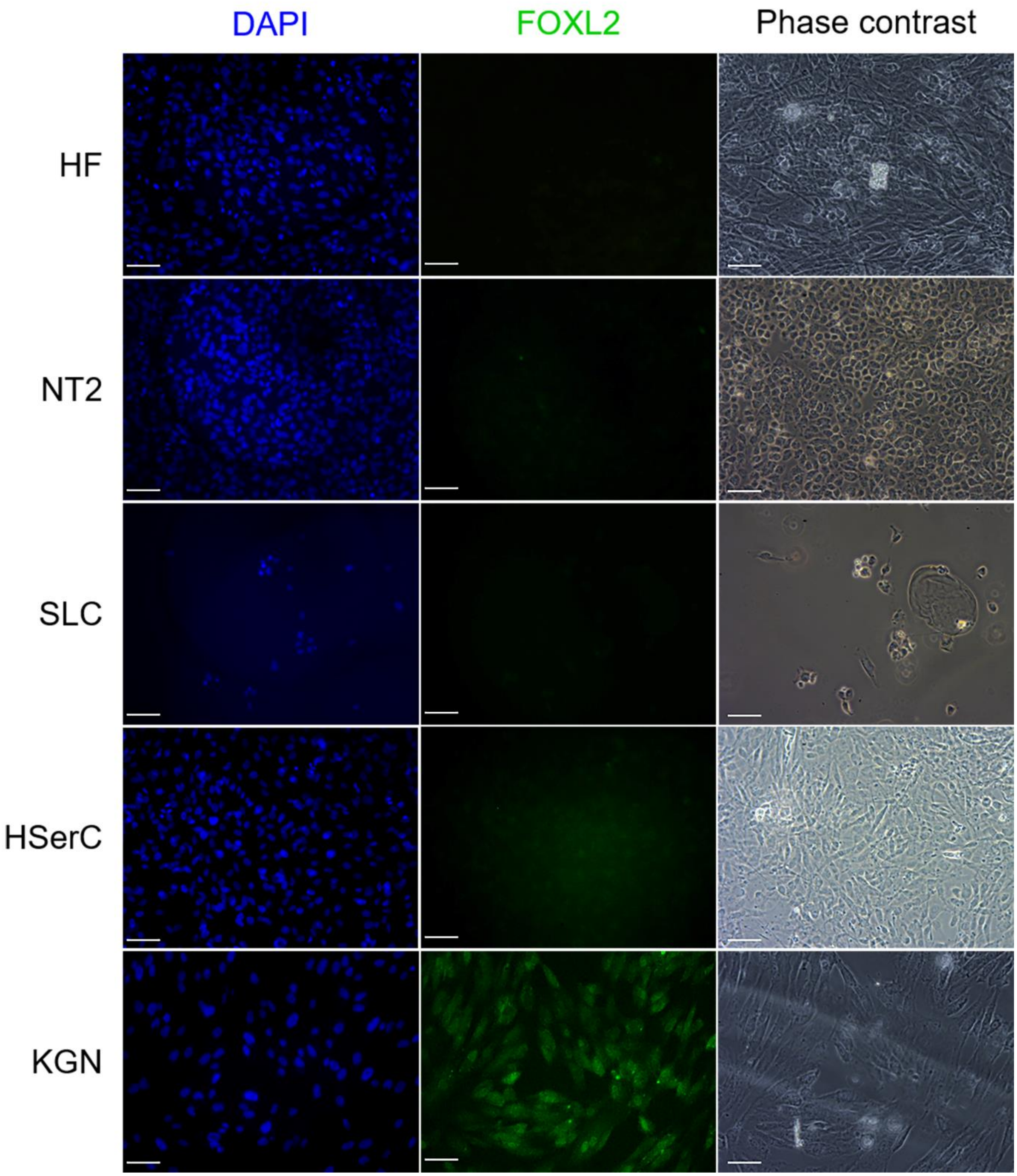

Supplementary figure S3: FOXL2 immunofluorescence staining analysis. Antibodies against FOXL2 were used. Mounting medium contains DAPI stain. SLCs were compared with NT2d1 cells andTM4 cells. KGN cells were used as a positive control for the expression of FOXL2. FOXL2 is absent or weakly expressed in Sertoli cell TM4 cells and SLCs but present in NT2d1 cells. HF: terminally differentiated fibroblasts, HSerCs: primary human Sertoli cells, NT2d1: NT2d1 cells, SLCs: Sertoli like cells. Scale bars 50 µm.
